# Nitrogen Fertilization and Solvents as Factors Modifying the Antioxidant and Anticancer Potential of *Arnica montana* L. Flower Head Extracts

**DOI:** 10.3390/plants12010142

**Published:** 2022-12-27

**Authors:** Danuta Sugier, Piotr Sugier, Joanna Jakubowicz-Gil, Urszula Gawlik-Dziki, Adrian Zając, Beata Król, Stanisław Chmiel, Magdalena Kończak, Mateusz Pięt, Roman Paduch

**Affiliations:** 1 Department of Industrial and Medicinal Plants, University of Life Sciences in Lublin, 15 Akademicka Street, 20-950 Lublin, Poland; 2 Department of Botany, Mycology and Ecology, Institute of Biological Sciences, Maria Curie-Skłodowska University, 19 Akademicka Street, 20-033 Lublin, Poland; 3 Department of Functional Anatomy and Cytobiology, Institute of Biological Sciences, Maria Curie-Skłodowska University, 19 Akademicka Street, 20-033 Lublin, Poland; 4 Department of Biochemistry and Food Chemistry, University of Life Sciences in Lublin, 8 Skromna Street, 20-704 Lublin, Poland; 5 Department of Hydrology and Climatology, Institute of Earth and Environmental Sciences, Maria Curie-Skłodowska University, Kraśnicka Av. 2d, 20-718 Lublin, Poland; 6 Institute of Earth and Environmental Sciences, Maria Curie-Skłodowska University, Kraśnicka Av. 2d, 20-718 Lublin, Poland; 7 Department of Virology and Immunology, Institute of Biological Sciences, 19 Akademicka Street, 20-033 Lublin, Poland; 8 Department of General and Pediatric Ophthalmology, Medical University of Lublin, 1 Chmielna Street, 20-079 Lublin, Poland

**Keywords:** *Arnica montana*, nitrogen fertilization, extraction methods, antioxidant activity, anticancer activity

## Abstract

*Arnica montana* L. is one of Europe’s endemic endangered medicinal plants, with diverse biological activities commonly used in medicine, pharmacy, and cosmetics. Its flower heads are a rich source of raw material, with antibacterial, antifungal, antiseptic, anti-inflammatory, antiradical, antioxidant, and antitumor properties. The objective of the present study was (i) to characterize the chemical composition of flower heads of *A. montana* plants cultivated under nitrogen fertilization, (ii) to identify the impact of the nitrogen fertilization and extraction method (water, ethanol) on the antioxidant activity of extracts, and (iii) to determine the role of different nitrogen doses applied during plant cultivation and different extraction methods in the anticancer activity of the extracts through analysis of apoptosis and autophagy induction in HT29, HeLa, and SW620 cell lines. The present study shows that nitrogen is a crucial determinant of the chemical composition of arnica flower heads and the antioxidant and anticancer activity of the analyzed extracts. Nitrogen fertilization can modify the composition of pharmacologically active substances (sesquiterpene lactones, flavonoids, essential oil) in *Arnicae flos*. The content of sesquiterpene lactones, flavonoids, and essential oil increased with the increase in the nitrogen doses to 60 kg N ha^−1^ by 0.66%, 1.45%, and 0.27%, respectively. A further increase in the nitrogen dose resulted in a decrease in the content of the analyzed secondary metabolites. Varied levels of nitrogen application can be regarded as a relevant way to modify the chemical composition of arnica flower heads and to increase the anticancer activity, which was confirmed by the increase in the level of apoptosis with the increase in fertilization to a level of 60 kg N ha^−1^. The fertilization of arnica plants with low doses of nitrogen (30 and 60 kg N ha^−1^) significantly increased the LOX inhibition ability of the ethanol extracts. The present study is the first report on the anticancer activity of *A. montana* water extracts, with emphasis on the role of water as a solvent. In further studies of factors modifying the quality of *Arnicae flos*, attention should be paid to the simultaneous use of nitrogen and other microelements to achieve synergistic results and to the possibility of a more frequent use of water as a solvent in studies on the biological activity of *A. montana* extracts.

## 1. Introduction

In the last few decades, the use of medicinal plants has been growing rapidly in the world due to the increasing demand for natural secondary metabolites, health products, and herbal drugs. Natural products and their derivatives are important sources of novel therapeutic substances, and new sources of secondary metabolites are widely being searched [1,2,3,4,5]. Therefore, a widespread demand for raw material originating from natural populations is observed [1,6,7]. A very good example is the mountain arnica (*Arnica montana* L.), a rare and endangered medicinal plant species that has been under pressure in the natural environment for years. *Arnica montana* is a perennial mountain plant endemic to Europe; it grows in Western European heathlands and in the pine forests of Eastern Europe [8,9,10,11,12]. The above-ground and underground parts of this special medicinal plant are characterized by the presence of valuable secondary metabolites, including sesquiterpene lactones, flavonoids, terpenoids, phenolic acids, and essential oils [13,14,15,16,17]. Its inflorescences, achenes, leaves, rhizomes, and roots are a rich source of raw material, with antibacterial, antifungal, antiseptic, anti-inflammatory, antiradical, antisclerotic, antioxidant, and antitumor properties [5,16,18,19,20,21,22,23,24].

The use of plant species representing functional food and medicinal plants as a source of valuable biologically active substances, especially those with antioxidant and anticancer activity, has been growing rapidly in the world [25,26,27]. In the last few years, the anticancer activity of metabolites, e.g., flavonoids and sesquiterpene lactones, characteristic also for mountain arnica, has been intensively studied [28,29,30,31,32,33]. Therefore, the introduction of this species to cultivation on a larger scale and the search for factors improving the yield, differentiation, and consequent diversification of the biological activity of secondary metabolites seem reasonable [34,35,36,37].

In the natural environment, accumulation of various SMs is frequently induced and modulated by a number of environmental factors simultaneously [38]. The content of secondary metabolites in *A. montana* inflorescences in the natural environment varies depending on the altitude, temperature during growth, type of vegetation, and differences in natural habitats [39,40,41]. In recent years, researchers have focused considerable attention on the determinants of the quantity and quality of mountain arnica raw material in field conditions, where environmental factors can be more efficiently controlled. In the different field experiments conducted recently, various biological and agrotechnical factors with a potential impact on the yield and chemical composition of raw material have been studied. These factors include plantation establishment, stages of plant development, different growth conditions, climatic conditions, reproduction type, planting period, or fertilization type [13,16,17,18,34,35,36,37,38,42]. However, the impact of these factors on the raw material quality and, in consequence, on its biological activity is rarely reported.

Among the macro- and micronutrients analyzed individually as a factor in different experiments on sustainable production of arnica raw material in field conditions, only the impact of foliar boron fertilization has been demonstrated [17]. In contrast, the impact of nitrogen as a basic nutrient has not been studied. This essential nutrient is one of the basic elements in the plant structure. It is involved directly or indirectly in the production of secondary metabolites. In the case of medicinal and aromatic plants, it can also influence the content and chemical composition of secondary metabolites [43,44,45,46]. Although *A. montana* has long been cultivated mainly for production of flower heads and many studies have been conducted to characterize this raw material, knowledge of the antioxidant and biological activity of water extracts of the species is still insufficient, as mainly methanol, ethanol, and acetone have been used as solvents [19,23,39,40,42,47,48]. In the case of arnica, total plant extracts have been observed to have better activity than pure compounds isolated from its flowers. In the literature, there are no results of analyses of the effect of water plant extracts on the antioxidant and anticancer activity; therefore, such studies are required [20]. Using different extraction methods, different quality of extracts and, in consequence, different antioxidant activity can be obtained [49]. A simultaneous use of different solvents, including rarely studied water extracts, can reveal the further antioxidant and anticancer potential of *Arnicae flos*. Therefore, the objective of the present study was (i) to characterize the chemical composition of flower heads derived from *A. montana* plants cultivated under nitrogen fertilization, (ii) to identify the impact of nitrogen fertilization and the extraction method (water, ethanol) on the antioxidant activity of extracts, and (iii) to determine the role of different nitrogen doses applied during plant cultivation and different extraction methods in the anticancer activity of the extracts through analysis of induction of cell death in HT29, HeLa, and SW620 cell lines.

## 2. Results

### 2.1. Chemical Characteristics of Raw Material

The content of flavonoids (Fs), sesquiterpene lactones (SLs), and essential oil (EO) in the flower heads of *A. montana* under nitrogen fertilization (NF) applied in the field experiment is presented in Table 1. It was found that the increase in the nitrogen dose from 0 to 60 (kg ha^−1^) resulted in an increase in the content of F (from 0.43% to 0.66%), SL (from 1.18% to 1.45%), and EO (from 0.24% to 0.27%) in the raw material. A further increase in the nitrogen dose (up to 90 kg N ha^−1^) resulted in a decline in the concentration of these metabolites in the flower heads.

### 2.2. Antioxidant Activity of Water and Ethanol Extracts

**Figure 1:**
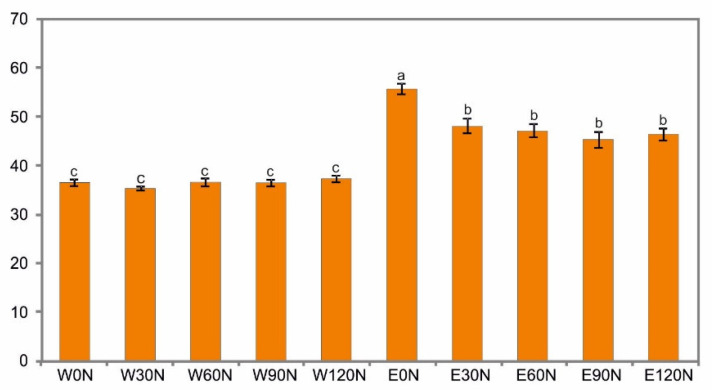
Total phenolic concentration (mg GAE/g DW) in water (W) and ethanol (E) flower head extracts of *A. montana* depending on the nitrogen doses; 0 N, 30 N, 60 N, 90 N, 120 N (kg ha^−1^). Values designated by the different letters are significantly different (*p* < 0.05).

The analysis of the content of polyphenolic compounds in the samples revealed that the extraction solvent had a significant influence on their amount (Table 2). The samples obtained with the use of ethanol had a statistically significantly higher content of total phenolic compounds (Figure 1).

**Figure 2:**
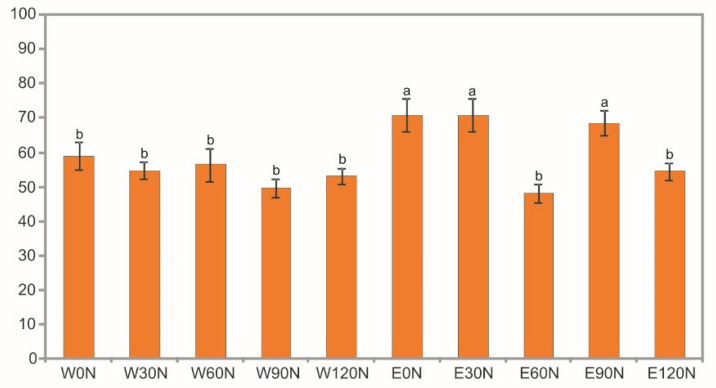
Chelating ability (%) of water (W) and ethanol (E) flower head extracts of *A. montana* depending on the nitrogen doses: 0 N, 30 N, 60 N, 90 N, 120 N (kg ha^−1^). Values designated by the different letters are significantly different (*p* < 0.05).

The two-way ANOVA results showed a statistically significant impact of the NF, the extraction method (water, ethanol), and their interaction on the chelating ability (CHEL). The water extracts exhibited a lower ability to chelate transition metal ions. In their case, no significant effect of the NF on the activity of the samples obtained from the plants was found. Equivocal results were obtained in the analysis of the activity of ethanol extracts. Compared to the samples obtained after water extraction, their activity was statistically significantly higher in the control sample and in the samples obtained from plants fertilized with doses of 30 and 90 kg N ha^−1^ and these values did not differ from each other (Figure 2).

**Figure 3:**
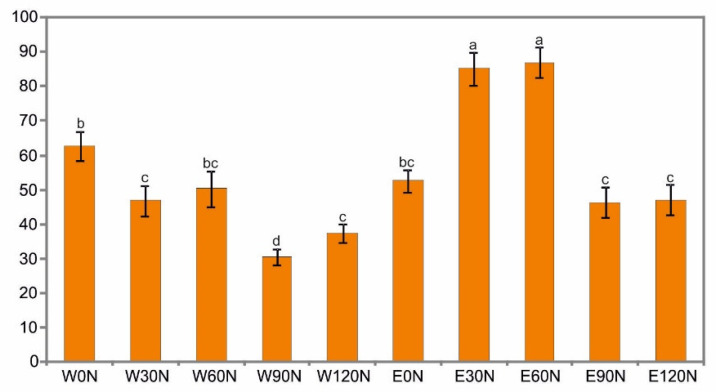
Lipoxygenase (LOX) inhibition ability (%) of water (W) and ethanol (E) flower head extracts of *A. montana* depending on the nitrogen doses: 0 N, 30 N, 60 N, 90 N, 120 N (kg ha^−1^). Values designated by the different letters are significantly different (*p* < 0.05).

Interesting results were obtained in the analysis of the ability of the extracts to inhibit lipoxygenase (LOX) activity. Both factors—the NF and the solvent—as well as their interaction had a significant impact on this parameter. The nitrogen fertilization caused a significant decrease in the activity of water-extractable LOX inhibitors. The lowest value was found for samples obtained from plants fertilized with a dose of 90 kg N ha^−1^. In turn, the NF at a dose of 30 and 60 kg N ha^−1^ resulted in a significant increase in the activity of ethanol-extractable LOX inhibitors (Figure 3).

**Figure 4:**
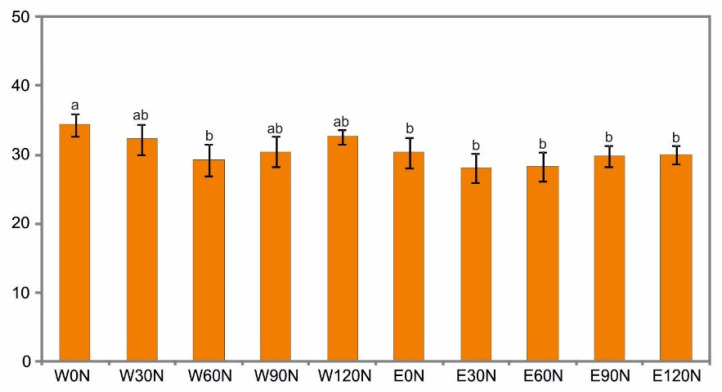
Free hydroxyl radical (OH) quenching ability (%) of water (W) and ethanol (E) flower head extracts of *A. montana* depending on the nitrogen doses: 0 N, 30 N, 60 N, 90 N, 120 N (kg ha^−1^). Values designated by the different letters are significantly different (*p* < 0.05).

The results of the statistical analyses showed a significant impact of the NF and the extraction method on the ability of the extracts to quench free hydroxyl radicals (OH). The effect of the interaction of the two factors was not confirmed statistically. The ethanol extracts showed a similar OH value. The water extracts from the unfertilized plants were characterized by slightly higher activity. There was no positive effect of the NF of the plants on the ability to neutralize free hydroxyl radicals (Figure 4).

### 2.3. Anticancer Activity of Water and Ethanol Extracts

To estimate the sensitivity of the HT29, HeLa, and SW620 cells to the treatment with the water and ethanol extracts derived from arnica flower heads, a staining method with dyes specific for apoptosis, necrosis, and autophagy, i.e., Hoechst 33342, propidium iodide, and acridine orange, respectively, was employed (Figure 1, Figure 2, Figure 3 and Figure 4). The results showed a statistically significant main effect of the NF, the extraction method, and the arnica extract concentration on apoptosis and necrosis in the studied cell lines (Table 3). In the case of the HT29 and HeLa cell lines, there was a statistically significant effect of the N × S, N × C, S × C, and N × S × C interactions on the apoptosis and necrosis induction. In the case of SW620, there was a significant effect only on cell apoptosis. The analyzed extracts had no effect on initiation of cell death in normal fibroblasts.

**Figure 5:**
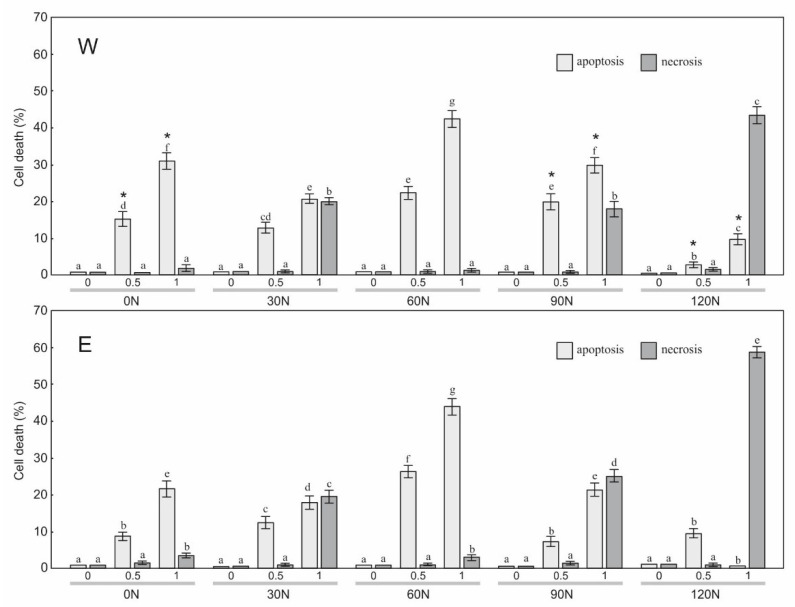
Level of apoptosis and necrosis induction in colon carcinoma HT29 cells treated with the water extract (W) and ethanol extract (E) (concentration: 0, 0.5, 1.0 μL/mL) from the flower heads of the *A. montana* plants. The values designated by the different letters are significantly different (*p* < 0.05). *—statistically significant difference (*p* < 0.05) between apoptosis values under the impact of W and E, at the same nitrogen dose and concentration.

The observations showed that the W and E variants added to the HT29 culture medium had a considerable effect on induction of cell death (Figure 5). A significant increase in the number of apoptotic cells was induced by W0N (15.3%) and E0N (8.7%) at a concentration of 0.5 µL/mL (Figure 5). The increase in the extract concentration to 1 µL/mL resulted in a further increase in the level of apoptosis to 31.0% in the case of W and to 21.7% in the case of E; simultaneously, necrosis at a level of 2.0% and 3.3%, respectively, was initiated. A significant (approx. 12%) increase in the number of apoptotic cells was induced by W30N and E30N at a concentration of 0.5 µL/mL (Figure 5). An increase in the extract concentration to 1 µL/mL resulted in a further increase in the level of apoptosis to 20.7% in the case of W and to 17.3% in the case of E and a simultaneous 20.0% and 19.1% increase in the level of necrosis, respectively. W60N and E60N added to the HT29 culture medium had a more promising effect on induction of cell death. A significant increase in the number of apoptotic cells was induced by W60N (22.3%) and E60N (26.3%) at a concentration of 0.5 µL/mL (Figure 5). The increase in the extract concentration to 1 µL/mL resulted in a further increase in the level of apoptosis to 42.7% in the case of W and to 44.0% in the case of E; simultaneously, necrosis at a level of 1.3% and 2.4%, respectively, was initiated.

**Figure 6:**
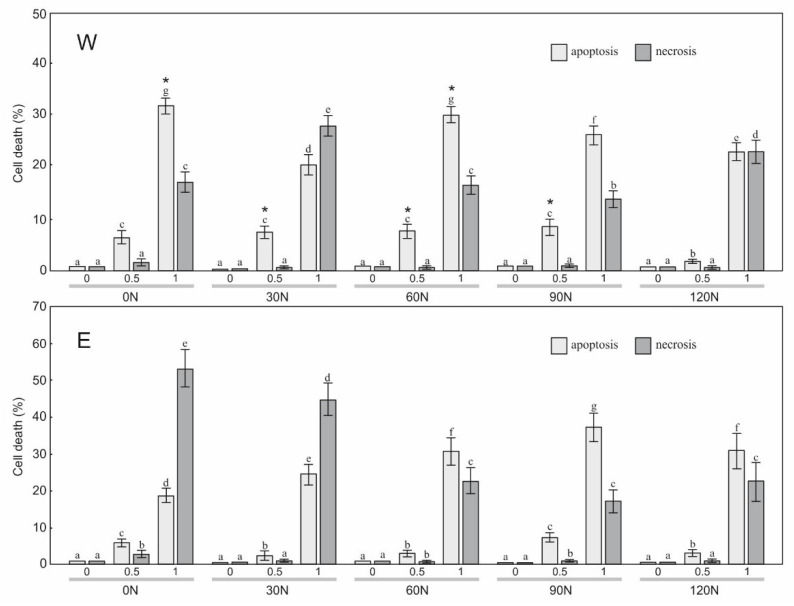
Level of apoptosis and necrosis induction in cervical carcinoma HeLa cells treated with the water extract (W) and ethanol extract (E) (concentration: 0, 0.5, 1.0 μL/mL) from the flower heads of the *A. montana* plants. Values designated by the different letters are significantly different (*p* < 0.05). *—statistically significant difference (*p* < 0.05) between apoptosis values under the impact of W and E at the same nitrogen dose and concentration.

The application of 0.5 µL/mL of W0N to the HeLa culture medium had a considerable effect on cell death, namely a significant 6.3% increase in the number of apoptotic cells was observed (Figure 6). A similar response was observed in the case of W30N, W60N, and W90N, i.e., the number of apoptotic cells increased to 7.7%, 7.3%, and 8.3%, respectively. A further increase in the W0N, W30N, W60N, and W90N concentrations to 1 µL/mL also resulted in a statistically significant increase in the level of apoptosis in a range of 20.3–31.3%, whereas necrosis exhibited a level of 13.6–27.7%. The application of 0.5 µL/mL of E0N, E30N, E60N E90N, and E120N to the HeLa culture medium had a considerable effect on cell death, namely there was a significant increase in the number of apoptotic cells: 5.7%, 2.3%, 7.0%, 7.3%, and 3.0%, respectively, accompanied by a low necrosis level (Figure 6). A further increase in the W0N, W30N, W60N, and W90N concentrations to 1 µL/mL also resulted in a statistically significant increase in the level of apoptosis in a range of 18.67–37.3%, whereas necrosis reached a level of 17.0–53.7%.

**Figure 7:**
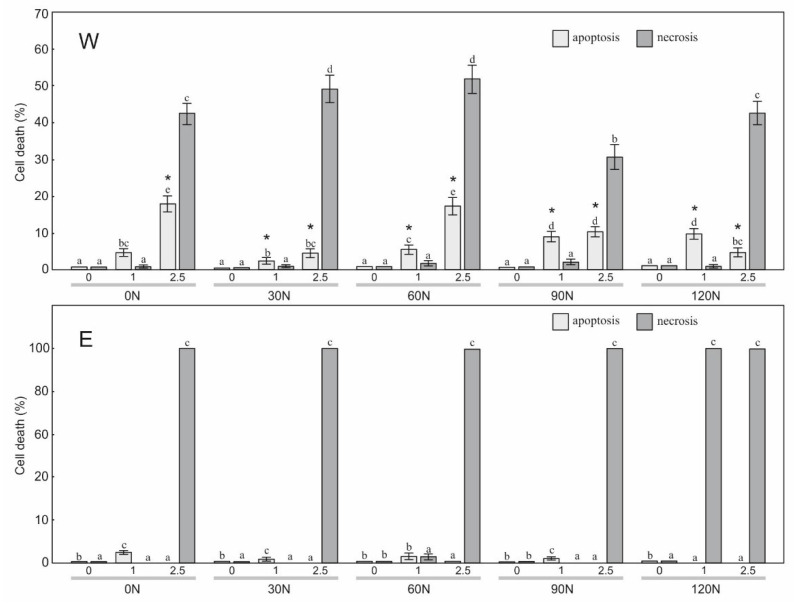
Level of apoptosis and necrosis induction in colon metastatic carcinoma SW620 cells treated with the water extract (W) and ethanol extract (E) (concentration: 0, 1.0, 2.5 μL/mL) from the flower heads of the *A. montana* plants. Values designated by the different letters are significantly different (*p* < 0.05). *—statistically significant difference (*p* < 0.05) between apoptosis values under the impact of W and E at the same nitrogen dose and concentration.

The weakest effect of the arnica extracts was found in the SW620 cell line. The microscopic observations demonstrated that W added to the SW620 culture medium had a considerable effect on induction of cell death at a concentration of 1.0 µL/mL (Figure 7). Apoptosis reached a level of 4.7%, 2.3%, 5.6%, 9.0%, and 9.7% in the W0N, W30N, W60N, W90N, and W120N variants, respectively. The increase in the extract concentration to 2.5 µL/mL resulted in a further increase in the level of apoptosis to 18.0%, 5.3%, 17.0%, 10.3%, and 5.0% for W0N, W60N, W90N, and W120N, respectively. Unfortunately, at the same time, the level of necrosis increased significantly. In the case of the E application, the level of apoptosis was very low at a concentration of 1 µL/mL. A further increase in the E0N, E30N, E60N, E90N, and E120N concentrations to 2.5 µL/mL resulted in an increase in the level of necrosis to 100%.

Interestingly, both the ethanol and water extracts had no effect on the level of autophagy in the control and in the treated cancer cells. Therefore, such results are not presented.

## 3. Discussion

### 3.1. Chemical Characteristics of Raw Material

In the present study, nitrogen fertilization did not affect the content of water-extractable polyphenolic compounds. In turn, lower TPC values were determined in all ethanol extract samples from plants fertilized with nitrogen, but no significant effect of the dose on changes in this content was found. Researchers suggest that the solvent polarity and plant species variety affect the extractability of polyphenols [50,51]. The higher total phenolic concentration in the ethanol versus water extracts was reported in a study of bearberry [49]. This thesis was also confirmed in the present study.

The present study showed that NF significantly modified the content of SL in the flower heads (Table 1). Their content in the mountain arnica raw material fertilized at a dose of 60 kg N ha^−1^ was over three-times higher than in the standards for *Arnicae flos* (minimum content of SL of 0.40%) specified in the Polish Pharmacopoeia XI [52] and was within the ranges reported by other authors: Douglas et al. [53]—from 0.66% to 0.94%, Seemann et al. [54]—from 0.40% to 1.55%, Aiello et al. [34]—from 0.45% to 1.51%, and Dall’Acqua et al. [55]—from 0.54% to 1.50%. In turn, substantially lower contents of SL were demonstrated by Ivanova et al. [56], who analyzed *Arnicae flos* from different wild populations, cultivated collections, botanical garden collections, and extracts purchased from a pharmacy store. The content of SL in the flower heads was significantly higher in the plots fertilized with 60 and 90 kg N ha^−1^ than in the control (Table 1). There are no available literature reports on the NF effect on the content of SL in the mountain arnica. A similar plant response to that described in the present study was reported by Olesińska et al. [45], who showed an increase in the content of SL with a simultaneous increase in nitrogen doses to 60 and 90 kg N ha^−1^ in meadow arnica flower heads.

Nitrogen participates in photosynthesis and respiration processes and in the production of primary and secondary metabolites. Various field experiments showed that different plants from the family Asteraceae fertilized with nitrogen contained the largest amounts of essential oil, which declined after applying nitrogen doses exceeding 75 kg N ha^−1^ [57]. The differences in the contents of biologically active/antioxidant compounds in arnica raw material, such as SL, phenolic acids, and flavonoids, are related to the different growth conditions and stages of plant development [42]. The role of nitrogen in the synthesis of SL has not been elucidated in *A. montana* to date. Differences in plant response to nitrogen have been demonstrated in the meadow arnica by Olesińska et al. [45] and in other plants represent the family Asteraceae [58,59].

Nitrogen fertilization also significantly modified the content of flavonoids in the flower heads (Table 1). Their content in the mountain arnica ranged from 0.43% in the control to 0.66% at a dose 60 kg N ha^−1^, which confirms previous findings reported by Sugier and Gawlik-Dziki [60]. There are no available literature reports on the NF effect on the content of flavonoids in the mountain arnica. Similar findings of the NF effect on the flavonoid content have been described in a study of *Arnica chamissonis*, where 60 kg N ha^−1^ was the most favorable nitrogen dose [45], and *Athrixia phylicoides* DC., where 75 kg N ha^−1^ was the most favorable nitrogen dose stimulating the synthesis of flavonoid compounds, with the highest increase in quercetin content [61]. In the cited studies, a further increase in the applied nitrogen doses led to a significant decrease in the concentration of flavonoids, as in the case of the mountain arnica. The content of sesquiterpene lactones and flavonoids in *Arnicae anthodium* is significantly dependent on the plant development phase [53,62]. The present results show that nitrogen may be another factor determining the SL content in arnica flower heads.

Nitrogen fertilization significantly modified the EO content in the raw material. The EO content in the arnica flower heads analyzed in the present study ranged from 0.22 to 0.27% (Table 1), which was higher than that reported in other literature data [13,17]. The content of this metabolite was also higher than its amount in arnica achenes (0.15–0.17%) [5] but lower than its content in arnica rhizomes and roots [16]. Literature data indicate that NF affects the EO content in various herbal raw materials [63,64,65,66]. A beneficial effect of NF was demonstrated also in the case of meadow arnica [45]. As shown in literature data, the EO content in *Arnicae flos* is significantly dependent on the maturity of flower heads [13], foliar boron application [17], and plant development phases [53,62]. The present results show that nitrogen may be another determinant of the EO content in arnica flower heads.

### 3.2. Antioxidant Activity

Antioxidants derived from medicinal plants have been increasingly investigated for their various health benefits [67]. The antioxidant potential of arnica flower heads has been studied with the use of numerous chemical assays and their very high antioxidant activity has been demonstrated [19,42,68]. The present study confirmed this finding. Each study conducted to date used different extraction methods and/or different extraction solvents. Mainly methanol, ethanol, and acetone were used as solvents in investigations focused on identification and characterization of new metabolites, determinants of variation in secondary metabolite profiles in *A. montana* flower heads, and antibacterial and antioxidant activity of arnica extracts [19,23,39,40,42,47,48]. Previous studies demonstrated that *Arnica* ethanol extracts exhibited high LOX and XO inhibitory activity and contained bioactive constituents useful in the treatment of LOX- and XO-induced diseases and inflammation [69]. The results presented in this study show that the type of solvents can determine antioxidant and biological activity, and the biological activity of water extracts differs from that of ethanol extracts. As shown in literature data, the differences in the contents of secondary metabolites in arnica raw material and their antioxidant activity were related to different plant parts, cultivation conditions, or stages of plant development [5,13,16,17,34,35,36,37,38,42]. The results presented in this study show that nitrogen and the type of solvent may be other factors determining the antioxidant activity of arnica flower head extracts.

### 3.3. Anticancer Activity

In the last few decades, much attention was paid in the literature to the antioxidant, antibacterial, and antifungal effects of arnica extracts [18,19,23,69,70]. However, the anticancer activity of mountain arnica raw material has scarcely been investigated. Considerable attention has been paid to the anticancer activity of EO extracted from arnica achenes, rhizomes, and roots [5,16]. Not only the plant part but also the plant age has an influence on the chemical composition of secondary metabolites and anticancer activity. Žitek et al. [24] reported apoptotic activity of *A. montana* flower head extracts against melanoma WM-266–4 cells. In turn, Flórez-Fernández et al. [68] showed cytotoxic properties against colorectal cancer HCT-116 and pancreatic adenocarcinoma PSN1 cells. It was clearly shown in the present study that NF determined the quality of the studied extracts and, in consequence, their anticancer activity in the case of all the analyzed cell lines. It was especially evident in the HT29 cell line, where the NF was shown to be a significant determinant of the chemical composition of the raw material and extracts and, in consequence, the induction of apoptosis in the HT29 cell line. The induction of apoptosis at a level of over 40% by the W and E variants at a concentration of 1 μL/mL and at a N dose of 60 kg ha^−1^ is a promising result. Previous studies showed that arnica EO extracted from achenes, rhizomes, and roots exerted an anticancer effect by induction of anaplastic astrocytoma MOGGCCM and glioblastoma multiforme T98G cell death [5,16]. As shown in the present study, anticancer activities (especially in the HT29 cell line) were exhibited by the *A. montana* flower head extracts as well. Therefore, this plant species can be included in the group with high anticancer potential and can be a source of molecules that can be exploited in medicine and the pharmaceutical industry.

The present results also demonstrated that the use of two types of extractants (water, ethanol) gives different effects in terms of anticancer activity. Different anticancer effects were obtained in the case of the HeLa cell line. Namely, the application of W caused a higher level of apoptosis than the E application in the case of two treatments, with no differences in the other cases. Although a very low level of apoptosis was found in the case of the SW620 cell line, the type of solvents was important here—the level of apoptosis was several-times higher in the W variant. This showed the possibility of an alternative use of water as a solvent in biological tests employed in the search for factors responsible for biological activity.

The extracts were not comprehensively chemically characterized in the present study; hence, we are unable to explain the differences in their biological activity. Nevertheless, it can be assumed that hydrophilic compounds, whose role has been demonstrated in the studies of other medicinal plants, may determine biological activity [49]. *Arnicae flos* is rich in quercetin, i.e., a distinctive bioactive flavonoid with well-documented anticancer activity [28,29,32,33]. Therefore, it is not excluded that this molecule, possibly with other hydrophilic compounds of W, can play a crucial role in the proapoptotic activity of water extracts. However, this hypothesis should be verified in separate studies.

## 4. Materials and Methods

### 4.1. Experimental Site Conditions

The research was conducted on an experimental plot (51°31′ N; 22°45′ E) located on podzolic soil characterized by moderate content of organic matter and phosphorus, low potassium content, very low magnesium levels, and very acidic pH. The field experiment was carried out in two-year-old arnica plantations in 2017–2018. The experiment was established in April 2017. Immediately after transfer from the plantation site (collection of the Department of Industrial and Medicinal Plants, University of Life Sciences in Lublin), seedlings with 6 to 8 leaves were planted at an 8–10 cm depth with 20 cm spacing and covered with soil. The 40 cm row spacing resulted in a density of 125,000 plants per ha^−1^. The first year of the experiment (2017) was regarded as a preliminary year (without nitrogen fertilization) due to the low yields of flower heads. The following year (2018) was regarded as the production year.

The experiment was set up on 5 m^−2^ plots using the randomized block method with three replications. In the experiment, five levels of nitrogen fertilization were applied: 0, 30, 60, 90, and 120 kg N per hectare. Nitrogen in the form of 34% ammonium nitrate was used in two doses: after the beginning of plant vegetation in spring and in the phase of inflorescence shoot formation. The same fertilization with phosphorus (single superphosphate 80 kg P ha^−1^) and potassium (potassium salt 120 kg K ha^−1^) was applied in autumn.

### 4.2. Plant Material

The raw material was harvested from 2-year-old *A. montana* individuals. Flower heads were collected successively from the entire plot in the full flowering phase (flower heads fully emerged, ligulate florets opened, tubular florets opened in up to half of the disc). After each harvest, fresh flower heads were weighed and dried in a drying room at 40 °C. After drying, the samples were weighed to determine the dry weight of the raw material. Next, the raw material intended for laboratory analyses was stored in paper bags at room temperature.

### 4.3. Chemical Analyses

#### 4.3.1. Sesquiterpene Lactones

Sesquiterpene lactones (SLs) were determined with the chromatographic method described in Polish Pharmacopoeia VIII [71]. Thus, 1 g of dried and ground *Arnicae anthodium* raw material was weighed on a laboratory scale and placed in a 250 mL round-bottom flask; next, 50 mL of a mixture of methanol and water in a 1:1 ratio was added. The contents of the flask were heated under a reflux in a water bath at 50–60 °C for 30 min with frequent shaking. The mixture was filtered through a cotton wool pad into a 500 mL round-bottom flask, and the filter residue with the cotton wool pad was flooded with a methanol–water mixture and heated in the same conditions for 30 min. The reaction mixture was filtered into a 500 mL round-bottom flask combining both filtrates. The extract was supplemented with 3 mL of the internal standard solution and evaporated to a volume of approx. 18 mL under reduced pressure. A methanolic solution of α-santonin (over 99% purity; Sigma-Aldrich, St. Louis, MO, USA) with a concentration of 0.001 g/mL was used as an internal standard. The concentrated extract was transferred onto a chromatographic column packed with diatomaceous earth (Sigma-Aldrich, St. Louis, MO, USA) in an amount of about 15 g. The solution was left on the column for approx. 20 min, and sesquiterpene lactones were eluted from 200 mL of a mixture of equal volumes of ethyl acetate and methylene chloride. The eluate was transferred into a 500 mL round-bottom flask and evaporated to dryness under reduced pressure using a vacuum evaporator. The dry residue was dissolved in 3 mL of 50% HPLC-grade methanol, and the resulting solution was passed under reduced pressure through a Captiva column filled with 02 µm grain size polypropylene for purification. The sample prepared in this way was used for HPLC analysis on a Varian ProStar apparatus equipped with a UV-ViS detector model 210. The results are presented as % of the sum of sesquiterpene lactones calculated as dihydrohelenalin tiglinate equivalents.

#### 4.3.2. Flavonoids

The content of flavonoids was determined with the spectrophotometric method specified by Polish Pharmacopoeia VI [72]. Thus, 0.5 g of ground raw material was placed in a 250 mL round-bottom flask and 20 mL of acetone, 2 mL of 25% hydrochloric acid, and 1 mL of a 0.5% urotropine (methenamine) solution were added and kept under a reflux in a MML 547 water bath from AJL Electronic for 30 min. The hydrolysate was filtered through a cotton-wool filter into a 100 mL volumetric flask. The precipitate and cotton wool were placed again into a 250 mL round-bottom flask, poured with 20 mL of acetone, and boiled again for 10 min. The extract was filtered into the same volumetric flask and supplemented with acetone to the mark. Next, 20 mL of the acetone extract was taken and placed in a pear-shaped funnel, 15 mL of distilled water was added, and the mixture was shaken. Then, 15 and 10 mL portions of ethyl acetate were added in portions and shaken each time. After separation of the water–acetone and acetate layers, the lower layer was poured out, while the upper (acetate) layer was collected for further analysis. The acetate layer was placed in a 50 mL volumetric flask and supplemented with ethyl acetate to the mark. In order to prepare solutions for the analyses, 10 mL of the acetate extract was collected into a 25 mL volumetric flask, and 2 mL of a 1% aluminum chloride solution was added and supplemented with a methanol and acetic acid mixture in a ratio of 19:1 to the required volume. The comparative solution was prepared analogously without the addition of the aluminum chloride solution. The solutions were allowed to stand for 45 min and their absorbance was measured at 425 nm on a HITACHI U-2900 spectrophotometer. The content of flavonoids (Fs) was expressed as quercetin equivalents.

#### 4.3.3. Essential Oil

The percentage of essential oils (EOs) was determined with the hydrodistillation method described in Polish Pharmacopoeia VI [72].

All chemical analyses were performed in triplicate.

### 4.4. Extract Preparation

To study the antioxidant activity, ethanolic and water extracts of *A. montana* flower heads were prepared. Thus, 250 mg of plant material was triple extracted using 5 mL of the solvent (99% ethanol or water) in a laboratory shaker for 30 min and then centrifuged. The combined extracts were used for further analyses [73].

#### 4.4.1. Total Phenolic Content (TPC)

The TPC of the extracts was determined with the method described by Singleton et al. [74] adapted for a microplate reader (Epoch 2 Microplate Spectrophotometer, BioTek Instruments). The total phenolic content was estimated as gallic acid equivalent (GAE) and expressed as mg GAE/g dry mass (DW).

#### 4.4.2. Metal-Chelating Activity (CHEL)

The metal-chelating activity (CHEL) was determined as described previously [75] using the following formula:

CHEL = [1 − (AS/AC)] × 100% (1)

where CHEL is the chelating ability, As is the absorbance of the sample, and Ac is the absorbance of the control.

#### 4.4.3. Inhibition of Lipoxygenase Activity (LOX)

The inhibition of LOX with linoleic acid as a substrate was measured spectrophotometrically with the method proposed by Axelrod et al. [76] adapted for a microplate reader (Epoch 2 Microplate Spectrophotometer, BioTek Instruments). The mixture contained 240 μL of 0.066 M phosphate buffer, 10 μL of a LOX solution, 10 μL of a pure substance solution, an ethanol extract solution, or digested extract. The reaction was started by adding 40 μL of 2.5 mmol/L linoleic acid. The change in the absorbance per minute at a wavelength of 234 nm was defined as a unit of LOX activity. All measurements were performed in three replicates.

#### 4.4.4. Ability to Scavenge Hydroxyl (OH•) Radicals

The OH• scavenging ability was determined according to Su et al. [77]. The scavenging activity was calculated using the following equation:

scavenging rate% = [1 − (A1 − A2)/Ac] × 100, (2)

where Ac is the absorbance of the control (without the tested sample), A1 is the absorbance of the tested sample addition, and A2 is the absorbance without sodium salicylate.

### 4.5. Cells and Culture Conditions

The study involved human carcinoma cell lines (cervical HeLa B, ECACC No 85060701; colon metastatic SW620, ATCC No CCL-227, and colon HT29, ATCC No HTB-38) cultured in RPMI 1640 medium (GIBCO BRL) supplemented with 5% fetal bovine serum (FBS) (GIBCO BRL) (*v*/*v*) and antibiotics (penicillin 100 U/mL, streptomycin 100 μg/mL, amphotericin B 0.25 μg/mL). Cells at a density of 1 × 10^6^ cells/mL were incubated at 37 °C in humidified atmosphere with 5% CO_2_.

### 4.6. Drug Treatment

Ethanol and water extracts of *A. montana* (final concentrations 1, 2, 5 µL/mL) were dissolved in DMSO and applied to the studied cancer cells for 24 h. HeLa, SW620, and HT29 cells incubated only with 0.01% of DMSO were used as controls; this concentration of DMSO had no effect on cell viability.

### 4.7. Detection of Apoptosis, Autophagy, and Necrosis

Apoptosis, autophagy, and necrosis were identified microscopically after staining with Hoechst 33,342 (Sigma), acridine orange (Sigma), and propidium iodide (Sigma) fluorochromes, respectively, as described previously [78]. A fluorescence microscope (Nikon E-800) was used for morphological analysis of dead cells. At least 1000 cells in randomly selected microscopic fields were counted. Each experiment was repeated three times, each with 1000 cells.

### 4.8. Statistical Analysis

After testing the data for normality (Shapiro–Wilk test) and homoscedasticity (Levene’s test), analysis of variance of different sets of data was performed followed by Tukey’s test. When necessary, log(x + 1) transformation was used before analysis. The results were expressed as means and SD, and the differences were considered significant at *p* < 0.05. The statistical analyses were carried out using the Statistica 12.0 software (Stat. Soft, Inc., Krakow, Poland).

## 5. Conclusions

The present study shows that nitrogen is a crucial determinant of the chemical composition of raw arnica flower heads and the antioxidant and anticancer activity of the analyzed extracts. Nitrogen fertilization can modify the composition of pharmacologically active substances (sesquiterpene lactones, flavonoids, essential oil) in *Arnicae flos*. The content of sesquiterpene lactones, flavonoids, and essential oil increased with the increase in the nitrogen doses to 60 kg N ha^−1^. Varied levels of nitrogen application can be regarded as a relevant way to modify the chemical composition of arnica flower heads and to increase the anticancer activity, which was confirmed by the increase in the level of apoptosis with the increase in fertilization to a level of 60 kg N ha^−1^. The fertilization of arnica plants with small doses of nitrogen (30 and 60 kg N ha^−1^) significantly increased the LOX inhibition ability of the ethanol extracts. In most cases (with the exception of the ability to neutralize free hydroxyl radicals), a dose of 60 kg N ha^−1^ and the selection of the extraction solvent were the differentiating factors. Ethanol was found to be a more effective extractant. The present study is the first report on the anticancer activity of *A. montana* water extracts, with emphasis on the role of water as a solvent. Nevertheless, further studies are required to explore their potential for future medicinal purposes. In further studies of factors modifying the quality of *Arnicae flos*, attention should be paid to the simultaneous use of nitrogen and other microelements to achieve synergistic results and to the possibility of a more frequent use of water as a solvent in studies on the biological activity of *A. montana* extracts.

## Figures and Tables

**Table 1:** Content of flavonoids (Fs), sesquiterpene lactones (SLs), and essential oil (EO) in the flower heads of *A. montana* depending on the nitrogen dose, 0, 30, 60, 90, 120—nitrogen doses (kg ha^−1^); values designated by different letters are significantly different (*p* < 0.05).

Nitrogen Dose (kg ha^−1^)	F (%)	SL (%)	EO (%)
0	0.43 ^d^ ± 0.027	1.18 ^cd^ ± 0.069	0.23 ^b^ ± 0.013
30	0.50 ^cd^ ± 0.027	1.26 ^bc^ ± 0.030	0.24 ^b^ ± 0.004
60	0.66 ^a^ ± 0.023	1.45 ^a^ ± 0.078	0.27 ^a^ ± 0.017
90	0.55 ^bc^ ± 0.044	1.37 ^ab^ ± 0.056	0.23 ^b^ ± 0.008
120	0.56 ^bc^ ± 0.033	1.09 ^d^ ± 0.084	0.22 ^b^ ± 0.014

**Table 2:** Impact of different types of solvent (water and ethanol) on the total phenolic concentration (TPC) and antioxidant activity. CHEL—chelating ability, LOX—lipoxygenase inhibition ability, OH—free hydroxyl radical, quenching ability.

	TPC (mg GAE/g DW)	CHEL (%)	LOX (%)	OH (%)
Nitrogen (N)	F = 0.70	F = 359.60	F = 38.33	F = 4.83
	*p* = 0.598	*p <* 0.001	*p <* 0.001	*p <* 0.01
Solvent (S)	F = 585.17	F = 526.86	F = 83.61	F = 20.19
	*p <* 0.001	*p <* 0.001	*p <* 0.001	*p <* 0.001
N × S	F = 1.90	F = 357.13	F = 20.85	F = 1.82
	*p* = 0.150	*p <* 0.001	*p <* 0.001	*p* = 0.165

**Table 3:** Effect of the main factors and their interactions on the level of apoptosis and necrosis. Result of multi-way analysis of variance (ANOVA). HT29—colon carcinoma, HeLa—cervical carcinoma, SW620—colon metastatic carcinoma.

Cell Line	HT29		HeLa		SW620	
	Apoptosis (%)	Necrosis (%)	Apoptosis (%)	Necrosis (%)	Apoptosis (%)	Necrosis (%)
Nitrogen (N)	F = 193.41	F = 128.60	F = 13.83	F = 65.61	F = 63.49	F = 2.58
	*p <* 0.001	*p <* 0.001	*p <* 0.001	*p <* 0.001	*p <* 0.01	*p <* 0.05
Solvent (S)	F = 50.43	F = 6.75	F = 6.16	F = 51.26	F = 3289.32	F = 17.48
	*p <* 0.001	*p <* 0.05	*p <* 0.05	*p <* 0.001	*p <* 0.01	*p <* 0.001
Concentration (C)	F = 2738.75	F = 1238.62	F = 1849.40	F = 18,008.19	F = 598.19	F = 1854.50
	*p <* 0.001	*p <* 0.001	*p <* 0.001	*p <* 0.001	*p <* 0.01	*p <* 0.001
N × S	F = 8.49	F = 2.060	F = 6.24	F = 15.71	F = 43.32	F = 0.661
	*p <* 0.001	*p* = 0.097	*p <* 0.001	*p <* 0.001	*p <* 0.01	*p* = 0.622
N × C	F = 58.77	F = 107.27	F = 10.44	F = 49.94	F = 29.10	F = 2.026
	*p <* 0.001	*p <* 0.001	*p <* 0.001	*p <* 0.001	*p <* 0.01	*p* = 0.058
S × C	F = 21.75	F = 7.89	F = 1.72	F = 218.27	F = 995.32	F = 27.89
	*p <* 0.001	*p <* 0.001	*p* = 0.187	*p <* 0.001	*p <* 0.01	*p <* 0.001
N × S × C	F = 36.74	F = 2.02	F = 6.63	F = 31.56	F = 5.43	F = 0.661
	*p <* 0.001	*p* = 0.059	*p <* 0.001	*p <* 0.001	*p <* 0.01	*p* = 0.724

## Data Availability

All the data are available from the corresponding author.
